# 95355 Potential Drug Therapy for Fragile X Tremor/Ataxia Syndrome

**DOI:** 10.1017/cts.2021.635

**Published:** 2021-03-31

**Authors:** Collis Brown, Tamaro Hudson, Sonya K. Sobrian

**Affiliations:** Howard University

## Abstract

ABSTRACT IMPACT: The ability to restore mitochondrial health in neurons derived from FXTAS patient-induced pluripotent stem cells by novel natural compounds is critically important to the management of patients experiencing this syndrome and other Fragile X associated disorders. OBJECTIVES/GOALS: The goal of this research is to assess the biological potency of MAM, NADA and MAM analogues’ neuroprotective capacity with respect to mitochondrial damage, and antioxidant properties that can restore mitochondrial health in patients with FXTAS. METHODS/STUDY POPULATION: To establish mitochondrial dysfunction, normal human cell lines and human induced pluripotent cells will be exposed to multiple concentrations of glucose/ glucose oxidase (GluOx) at several time points to induce varying intensities of oxidative stress. The degrees of oxidative stress will be measured by apoptosis and mitochondrial reactive oxygen species (ROS) production. N-arachidonoyldopamine (NADA), macamides (MAM) and its analogue compounds, effective against oxidative damage in mitochondria, will be used to rescue glucose oxidase induced oxidative damage in both cell lines. To test the ability of these drugs to restore mitochondrial health, cell viability and cellular superoxide production will be assessed by propidium iodide and the MitoSox fluorescence assay, respectively. RESULTS/ANTICIPATED RESULTS: We anticipate that GluOx at varying concentrations and time points will proportionally increase levels of apoptosis and mitochondrial ROS, reflective of mitochondrial damage, with the most severe dysfunction occurring at the maximum dose of 40µM and the longest duration of 72-hr exposure. Moreover, administration of NADA, MAM, and MAM analogues at seven concentrations, ranging from 10-8 to 10-5 M in half-log increments, will successfully treat the oxidative defects induced in the cell lines by decreasing apoptosis, and superoxide production, and increasing cell viability. DISCUSSION/SIGNIFICANCE OF FINDINGS: This research allows for the development of an in vitro neuronal model of FXTAS, lends flexibility to testing therapeutics, and expands the discovery of mitochondrial biomedical markers for the syndrome. Data generated should inform mechanistic studies of the relationship between mitochondrial damage and FXTAS-induce neurodegeneration.

